# Recent Experiences with the Schäfer Method of Artificial Respiration

**Published:** 1909-07-31

**Authors:** Clinton T. Dent

**Affiliations:** Senior Surgeon to St. George's Hospital and Chief Surgeon to the Metropolitan Police


### Hospital Clinics.

### RECENT EXPERIENCES WITH THE SCHÄFER METHOD OF ARTIFICIAL RESPIRATION.

By CLINTON T. DENT, M.C., F.R.C.S.; Senior Surgeon to St. George's Hospital and Chief Surgeon to the Metropolitan Police.

(Abstract of a lecture delivered at the Polyclinic.)

About the actual technique of the Schäfer method of artificial respiration I need not say more than a few words. It is founded on the principle of utilising abdominal respiration, and it has some features in common with the method introduced and demonstrated many years ago in this country by Dr. Benjamin Howard, of, I think, New York. But Schäfer's method differs in this, that it depends entirely on the fact that in ordinary tranquil respiration the diaphragm sinks with inspiration, and as it sinks pushes forward the epigastrium. If, therefore, you compress this portion of the belly, you push the viscera back against the diaphragm, drive it upwards, and expel air from the lungs. Schäfer insists on this principle, and has no desire to compress the thorax at all, though practically the thorax is to some extent compressed. He has shown that compression of this part of the abdomen causes a large exchange of air, larger, he thinks, than can be obtained by other methods, including Silvester's. Professor Keith and others, however, doubt this latter conclusion.

I should like to show you first the Howard method. Howard put a large pad under the shoulders, and bent the head right back until it was at right angles to the trunk: in this position the tongue is drawn forwards, and the glottis is necessarily open. Then he got astride of the patient and forcibly compressed the thorax. He grasped the lower ribs, and by so doing to some extent made pressure on the abdominal viscera and pushed them against the diaphragm.

Professor Schäfer also at first advised a pad under the back, but he afterwards abandoned it as not only unnecessary, but positively injurious. He places the patient prone with his face turned to one side, and, kneeling either at the side of him or astride—astride is very much better—he places the palms of the hands on the loins as low down as possible: the thumbs should be parallel on either side of the spine. In giving instruction to the police we insist that the palms of the hands should not touch, but be placed just above the iliac crests: the fingers just reach the lowest ribs. All that need be done by the operator is to press and relax alternately by leaning forward on to the outstretched arms, waiting a couple of seconds, and then without removing the hands throwing the body back on to his haunches. This pressure causes the expiration that would normally be produced by the natural resilience of the abdominal wall. It must be noted that in an apparently drowned person (or one actually dead) the abdominal wall is much less elastic and resilient than in normal individuals. When the weight of the body is thus thrown on to the arms, a pressure of forty, fifty, or sixty pounds is exerted, which is quite enough: this is much less likely to do any harm than is the Howard method.

Such briefly is the Schäfer method in its perfected form. Repeated experiments have been made on the living human subject as well as upon dogs and other animals. The first public body to take up this new method (or, if you please, this new modification of an existing method) was the Life Saving Society, and great credit is due to them for it. They adopted it in 1905. Some time later, dissatisfied with the methods of Silvester and Marshall Hall, I was anxious to see whether this simpler method might do for the Metropolitan Police. Since anybody holding any office in a Public Department is, practically speaking, a target to be shot at, I thought it wise, before introducing it, to take other opinions, and the Royal Society of Medicine, at my request, went into the matter. They recommended it for the apparently drowned, and it has now been officially adopted for the Metropolitan Police. Although it has been in use only for a few months, and no one can as yet form an opinion of its full value, enough has already been seen to show that the change is undoubtedly a judicious one.

Every new method introduced upon the medical stage seems to pass through certain definite vicissitudes. At the outset full of promise, it embarks on a career of great success until critics or the introducers of new measures fall upon it. If it survives these onslaughts, the originator is often the next to fall foul of his own protégé. He will point out how he has improved on the original by his later modifications. This has been especially the case in the treatment of the apparently drowned. A fair criticism of the Schäfer method is that the original experiments were made upon living individuals, and even on those who were prepared by a series of deep inspirations; not upon the apparently drowned. The difference is extremely great: but as a matter of fact Schäfer had previously experimented on dogs which had been immersed for varying periods.

My chief object this afternoon is to put before you the experience of cases actually treated, and to show how far this experience proves the method to be good practically. It is, I think, certainly the simplest and least fatiguing method yet devised, and certainly the best when there is only one person to carry out the resuscitation.

There have been many cases of apparent drowning under treatment, for a fair proportion of the cases with which the police have to do are of attempted suicide by jumping off bridges. It is curious how a particular bridge becomes fashionable for this purpose for a time: at one time Waterloo Bridge was almost constantly selected, though it is much easier to jump off Westminster Bridge. Sometimes the intending suicide selects the Embankment, and there is another curious point in connection with this. Between Westminster and Waterloo Bridges such cases were very common, and are now extremely rare. I asked one of the police, who has been on Waterloo Pier for twenty years, if he could explain this. He said in the old days there was no traffic on the Embankment, which was almost deserted at night: but since the introduction of tramcars and the electric light suicides have become very rare. People still go down to the Embankment with the intention of committing suicide, but they do not carry it out. With regard to those who jump from the bridges, many are not drowned, but are fatally injured by striking against the buttresses as they fall.

The first case treated by the Schäfer method was that of a man found floating face downwards in the Regent's Park Canal: he was still warm when removed, though it was quite uncertain how long he had been immersed. The Schäfer method was employed for 30 minutes, but without success. I never heard of any other system of artificial respiration yet which started with a failure as this did. At the post-mortem the lungs were distended to their fullest extent: air and water exuded from them on pressure. It is, therefore, certain that air had got well into the lungs. As usual, all the abdominal vessels were engorged with blood, and this raises an important question. Whether you should in these cases be too instant in attempting to empty the veins of the portal system is somewhat a moot point. If you do, you are overfilling what is already full. In the Schäfer method you undoubtedly stimulate the heart's action.

In Professor Schäfer's instructions, as printed for the use of the police, it is laid down that no time is to be wasted in removing clothing: but I think myself that certainly some loosening of the clothing is desirable. In the case I have mentioned the medical man who was summoned and watched the artificial respiration being performed noted that the patient's thick clothing seemed to him to hamper the effective application of the pressure. It is to be remembered that viscera, such as the liver, which are engorged with blood are friable and may be ruptured by excessive force. Belts, and those mysterious garments called corsets, may interfere materially with the descent of the diaphragm, and it seems quite clear to me that if any assistant is at hand he should devote himself to loosening the garments.

The next case was that of a man of over sixty, who was immersed for about five minutes, perhaps rather more, perhaps rather less, in bitterly cold weather. This again was a failure. I mention it because it occurred, and because the Schäfer method was employed actually in the boat by which he was rescued: this is a great advantage of the new method, which can easily be performed in quite a small boat. Other cases of apparent drowning have been successfully revived.

It has been suggested to me by one or two of the divisional surgeons that the method is more adapted for thin persons and for men rather than for women, but with this I do not agree. If the method is used properly the result will be as good in the stout as in the emaciated, and in women as in men. Mr. Henry, the Secretary of the Life Saving Society, has remarked that if a stout and an emaciated person are immersed for the same length of time, the former has much less chance of recovery than the latter. Another suggestion is—and Silvester himself made much of it only a year or so before his death—that the astride position is improper. Even if it were, that surely does not matter much when it is a case of life or death. I fail myself to see anything improper at all about it. Still, the point has been raised, and it is worth noting that one can do the movements and exert the pressure just as effectively, though less easily, without getting astride of the patient.

Another case I should like to mention occurred at Southampton. For ten minutes the Silvester method was used without success: then came a police sergeant, who succeeded with the Schäfer method. I do not claim this as any proof of the superiority of the latter, for Silvester's method cannot be said to have failed. But the police themselves think the new method is superior.

It is also interesting to consider cases where drowning is not in question. There is the case of a man who was overpowered by heat. The Schäfer method was employed for a few minutes with success, but it was used with a big pad under the thorax: it was therefore not really the Schäfer method at all, but Howard's method reversed. The medical man who did it thought highly of the result. Another case was one of coal-gas poisoning, but since the two bodies were cold and rigor mortis present no recovery was possible, and it is not surprising to hear that the Schäfer method failed after an hour and a half. It was, however, noted that the expiratory movements produced were enough to blow out a lighted match. The method is certainly also applicable to, and has been successfully employed in, strangulation by hanging. Another curious case is that of a boy who was struck a very severe blow on the side of the neck by the shaft of a cart. Respiration ceased, and it was at first thought that he had broken his neck. However, he recovered under a combination of the Schäfer method and oxygen, and was probably suffering from the effects of the blow upon the pneumogastric and other main nerves of the neck.

One more case: a woman attempted to drown two small children in a bath. The Silvester method was used by the medical man called in, because he thought the Schäfer method should not be employed for children. But it happened a few days ago that a constable was attracted to a burning house from which a man and woman had escaped, leaving some children inside. Taking a series of deep breaths, and keeping a wet cloth over his mouth and nose the constable bravely entered the burning house and rescued a small child. A passer-by followed his example and rescued another. Both children were deeply asphyxiated, and the Schäfer method was pursued for 30 and 40 minutes respectively before breathing recommenced. That case shows conclusively, to my mind, that the method may be used safely and advantageously in the case of children.

These are only scattered notes from our present records, but I think they show that the method is applicable to every sort of case, and that Professor Schäfer's claims for it are not only well-founded but stand the practical test of experience.

